# First person – Lena Menzel

**DOI:** 10.1242/bio.062590

**Published:** 2026-04-24

## Abstract

First Person is a series of interviews with the first authors of a selection of papers published in Biology Open, helping researchers promote themselves alongside their papers. Lena Menzel is first author on ‘ An ARVC-5 *Drosophila* knock-in model reveals new functions of Tmem43 in lipid homeostasis’, published in BiO. Lena is a PhD student in the lab of Achim Paululat at the University of Osnabrück, Germany, investigating ways to utilise our *Drosophila* model to translate our findings to humans, which may ultimately help real-world patients.

**Figure:**
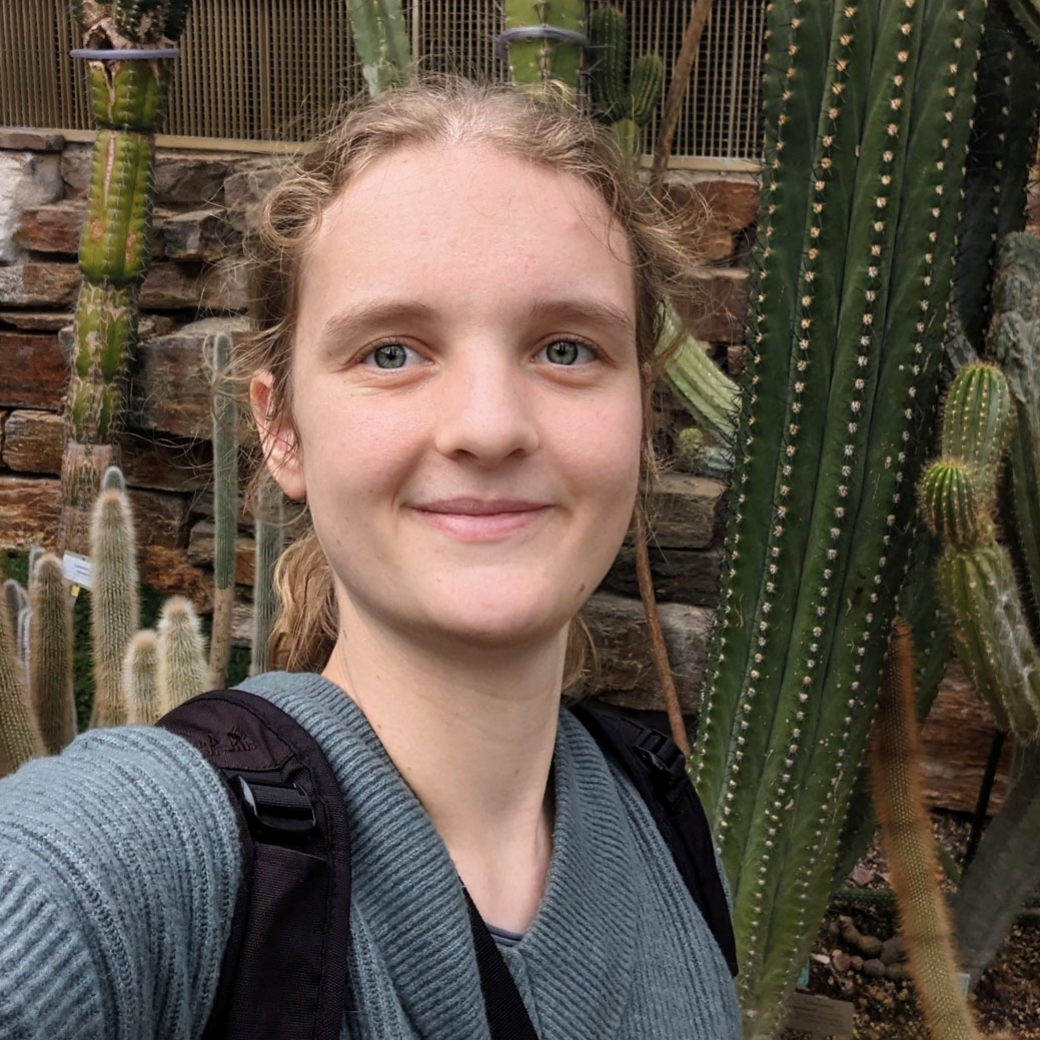
Lena Menzel

**Describe your scientific journey and your current research focus**

I have been part of the Zoology department of the University of Osnabrück (Paululat Lab) for one year now, where I work with *Drosophila melanogaster*, which you may know as the annoying fruit fly taking over your bananas. At first glance, they seem so different to us, yet almost 70% of hereditary diseases are conserved in *Drosophila*, which allows me to study complex conserved mechanisms in an accessible system. This includes *TMEM43*, where a single mutation (p.S358 L) can lead to a severe form of arrhythmogenic right ventricular cardiomyopathy (ARVC). This is a heart disease that causes sudden cardiac death (SCD) and has no therapy currently available. We work with overexpression lines and a recently established knock-in line to study the function of Tmem43 and the role of its mutation in ARVC-5. We did this to be as close as possible to the situation in humans, which is quite exciting for us.

**Who or what inspired you to become a scientist?**

Since childhood, I have been fascinated by biology in general. Why do children resemble their parents? What has an influence on who we are? What differentiates us from animals? In secondary school, I had an inspiring biology teacher who further nurtured my passion, especially for genetics. Before I knew it, I ended up studying biology and working with *Drosophila*, one of THE model organisms for genetics. I love studying the unknown and the thought that my work might one day help actual patients motivates me even more.

**How would you explain the main finding of your paper?**

This work establishes an additional *Drosophila* model for ARVC-5: the knock-in. Our previously established model overexpresses the mutated Tmem43 tissue specifically. While this provides a lot of advantages for experimental design and results in strong phenotypes, this does not mirror the human situation perfectly. This is why, with this work, we wanted to change that. Here we introduced the conserved p.S333L mutation endogenously, resulting in native protein levels. While heart parameters in these flies are not affected under normal conditions, these animals nevertheless show most of the characteristic Tmem43p.S333L-overexpression phenotypes, including reduced life span, reduced size and weight, and an increased number of damaged mitochondria. Furthermore, our models were used to show dose-dependent proteomic and lipidomic remodelling, which nicely fits into existing literature from other model organisms.

The establishment of the knock-in, which mirrors the human situation, allows us to further research the role of Tmem43 mutation in this devastating disease in a simple and inexpensive model organism

**What are the potential implications of this finding for your field of research?**

The establishment of the knock-in, which mirrors the human situation, allows us to further research the role of Tmem43 mutation in this devastating disease in a simple and inexpensive model organism. This was our first step on the endogenous level, which now allows us to alter the conditions and get as close as we can to the human phenotype. While there are some hypotheses, the exact function of the wild-type Tmem43 and the effect of the mutation remain elusive, so that is something that remains open.

**Figure:**
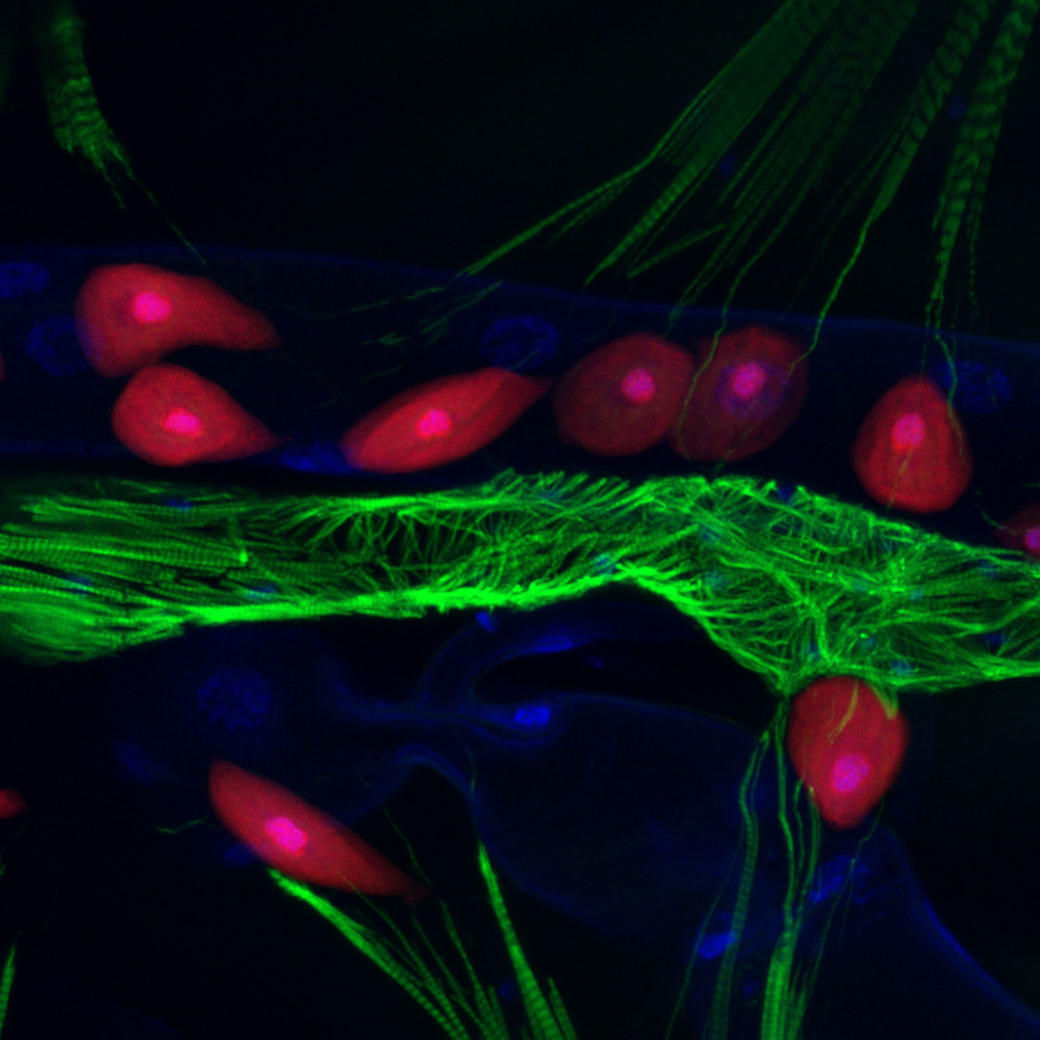
**The *Drosophila* heart – so different, yet so similar.** The image shows a section of the heart of a prepared third instar larva expressing *handC*>mCherry NLS, stained with FITC and DAPI. While, different to human, the *Drosophila* heart is a linear muscular tube, and many mechanisms are conserved across the species.

**Which part of this research project was the most rewarding?**

We are fortunate enough to have a collaboration with the HDZ in Bad Oeynhausen, where our partners are working with human cells. Together, we have the aim of identifying the mechanism and potential therapeutic approaches. I mean it is quite the motivation to know that you are working on something that people suffer from every day and that, in some sense, your research might one day have a direct effect on them.

**What do you enjoy most about being an early-career researcher?**

I enjoy learning what it means to be a researcher. It is this fascinating and exciting phase where one does things for the first time. The first own project and own experiments. The first paper and first time being at a conference, showing a poster, presenting your data. You learn a lot and become part of this huge community, with other early career researchers like yourself but also senior scientists, who show you there is so much more to learn, to explore.

**What piece of advice would you give to the next generation of researchers?**

You will fail. Not once. Not twice. But again, and again. And it's okay! This is where you have to remember that you are not alone. This is part of science. I did. Others did. We all have gone through it. Many experiments won't work on the first try. Some not after the tenth… You may be thinking of giving up. Don't! This is all part of the journey. Take your time to reorganise and talk to others. There is no shame in asking. And who knows? They may have an idea that leads to the breakthrough. And nothing – really nothing – is better than the feeling when your hard work finally pays off. You can do it!

**What's next for you?**

I am just getting started. As I am still in the early stages of my PhD, there are many open questions I want to address and many experiments to do. I will further work on this project and further unravel the mystery of Tmem43, hopefully adding a small puzzle piece to approaching a treatment for this mutation.
